# A comparison of random forest and its Gini importance with standard chemometric methods for the feature selection and classification of spectral data

**DOI:** 10.1186/1471-2105-10-213

**Published:** 2009-07-10

**Authors:** Bjoern H Menze, B Michael Kelm, Ralf Masuch, Uwe Himmelreich, Peter Bachert, Wolfgang Petrich, Fred A Hamprecht

**Affiliations:** 1 Interdisciplinary Center for Scientific Computing (IWR), University of Heidelberg, Heidelberg, Germany; 2 Computer Science and Artificial Intelligence Laboratory (CSAIL), Massachusetts Institute of Technology, Cambridge/MA, USA; 3 Micro-Biolytics GmbH, Esslingen, Germany; 4 Biomedical NMR Unit, Department of Medical Diagnostic Sciences, KU Leuven, Leuven, Belgium; 5 Department of Medical Physics in Radiology, German Cancer Research Center, Heidelberg, Germany; 6 Department of Astronomy and Physics, University of Heidelberg, Heidelberg, Germany; 7 Roche Diagnostics GmbH, Mannheim, Germany

## Abstract

**Background:**

Regularized regression methods such as principal component or partial least squares regression perform well in learning tasks on high dimensional spectral data, but cannot explicitly eliminate irrelevant features. The random forest classifier with its associated Gini feature importance, on the other hand, allows for an explicit feature elimination, but may not be optimally adapted to spectral data due to the topology of its constituent classification trees which are based on orthogonal splits in feature space.

**Results:**

We propose to combine the best of both approaches, and evaluated the joint use of a feature selection based on a recursive feature elimination using the Gini importance of random forests' together with regularized classification methods on spectral data sets from medical diagnostics, chemotaxonomy, biomedical analytics, food science, and synthetically modified spectral data. Here, a feature selection using the Gini feature importance with a regularized classification by discriminant partial least squares regression performed as well as or better than a filtering according to different univariate statistical tests, or using regression coefficients in a backward feature elimination. It outperformed the direct application of the random forest classifier, or the direct application of the regularized classifiers on the full set of features.

**Conclusion:**

The Gini importance of the random forest provided superior means for measuring feature relevance on spectral data, but – on an optimal subset of features – the regularized classifiers might be preferable over the random forest classifier, in spite of their limitation to model linear dependencies only. A feature selection based on Gini importance, however, may precede a regularized linear classification to identify this optimal subset of features, and to earn a double benefit of both dimensionality reduction and the elimination of noise from the classification task.

## Background

The high dimensionality of the feature space is a characteristic of learning problems involving spectral data. In many applications with a biological or biomedical background addressed by, for example, nuclear magnetic resonance or infrared spectroscopy, also the number of available samples *N *is lower than the number of features in the spectral vector *P*. The intrinsic dimensionality *P*_*intr *_of spectral data, however, is often much lower than the nominal dimensionality *P *– sometimes even below N.

### Dimension reduction and feature selection in the classification of spectral data

Most methods popular in chemometrics exploit this relation *P*_*intr*_* < P *and aim at regularizing the learning problem by *implicitly *restricting its free dimensionality to *P*_*intr*_.

(Here, and in the following we will adhere to the algorithmic classification of feature selection approaches from [1], referring to regularization approaches which explicitly calculate a subset of input features – in a preprocessing, for example – as *explicit *feature selection methods, and to approaches performing a feature selection or dimension reduction without calculating these subsets as *implicit *feature selection methods.) Popular methods in chemometrics, such as principal component regression (PCR) or partial least squares regression (PLS) directly seek for solutions in a space spanned by ~*P*_*intr *_principal components (PCR) – assumed to approximate the intrinsic subspace of the learning problem – or by biasing projections of least squares solutions towards this subspace [2,3], down-weighting irrelevant features in a constrained regression (PLS). It is observed, however, that although both PCR and PLS are capable learning methods on spectral data – used for example for in [4] – they still have a need to eliminate useless predictors [5,6]. Thus, often an additional *explicit *feature selection is pursued in a preceding step to eliminate spectral regions which do not provide any relevant signal at all, showing resonances or absorption bands that can clearly be linked to artefacts, or features which are unrelated to the learning task. Discarding irrelevant feature dimensions, though, raises the question of how to choose such an appropriate subset of features [6-8].

Different univariate and multivariate importance measures can be used to rank features and to select them accordingly [1]. Univariate tests marginalize over all but one feature and rank them in accordance to their discriminative power [9,10]. In contrast, multivariate approaches consider several or all features simultaneously, evaluating the joint distribution of some or all features and estimating their relevance to the overall learning task. Multivariate tests are often used in wrapper schemes in combination with a subsequent classifier (e.g. a global optimization of feature subset and classifier coefficients [11]), or by statistical tests on the outcome of a learning algorithm (e.g. an iterative regression with test for robustness [12,13]). While univariate approaches are sometimes deemed too simplistic, the other group of multivariate feature selection methods often comes at unacceptably high computational costs.

### Gini feature importance

A feature selection based on the random forest classifier [14] has been found to provide multivariate feature importance scores which are relatively cheap to obtain, and which have been successfully applied to high dimensional data, arising from microarrays [15-20], time series [21], even on spectra [22,23]. Random forest is an ensemble learner based on randomized decision trees (see [24] for a review of random forests in chemometrics, [14] for the original publication, and [25-28] for methodological aspects), and provides different feature important measures. One measure is motivated from statistical permutation tests, the other is derived from the training of the random forest classifier. Both measures have been found to correlate reasonably well [28]. While the majority of the prior studies focused on the first, we will focus on the second in the following.

As a classifier, random forest performs an *implicit *feature selection, using a small subset of "strong variables" for the classification only [27], leading to its superior performance on high dimensional data. The outcome of this implicit feature selection of the random forest can be visualized by the "Gini importance" [14], and can be used as a general indicator of feature relevance. This feature importance score provides a relative ranking of the spectral features, and is – technically – a by-product in the training of the random forest classifier: At each node *τ *within the binary trees *T *of the random forest, the optimal split is sought using the Gini impurity *i*(*τ*) – a computationally efficient approximation to the entropy – measuring how well a potential split is separating the samples of the two classes in this particular node.

With  being the fraction of the *n*_*k *_samples from class *k *= {0,1} out of the total of *n *samples at node *τ*, the Gini impurity *i*(*τ*) is calculated as



Its decrease Δ*i *that results from splitting and sending the samples to two sub-nodes *τ*_*l *_and *τ*_*r *_(with respective sample fractions  and ) by a threshold t_*θ *_on variable *θ *is defined as



In an exhaustive search over all variables *θ *available at the node (a property of the random forest is to restrict this search to a random subset of the available features [14]), and over all possible thresholds t_*θ*_, the pair {*θ*, t_*θ*_} leading to a maximal Δ*i *is determined. The decrease in Gini impurity resulting from this optimal split Δi_*θ *_(*τ*, *T*) is recorded and accumulated for all nodes *τ *in all trees *T *in the forest, individually for all variables *θ*:



This quantity – the Gini importance *I*_*G *_– finally indicates how often a particular feature *θ *was selected for a split, and how large its overall discriminative value was for the classification problem under study.

**Figure 1:**
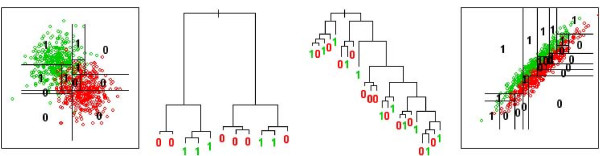
**Decision trees separating two classes: Classification problem with uncorrelated features (left), and a distorted version resulting from an additive noise process (right)**. The said process induces correlation by adding a random value to both features, thus mimicking the acquisition process of many absorption, reflectance or resonance spectra (see Methods section). Growing orthogonal decision trees on such a data set – shown on the right – results in deeply nested trees with complex decision boundaries. (Both trees not grown to full depth for visualization purposes).

When used as an indicator of feature importance for an explicit feature selection in a recursive elimination scheme [1] and combined with the random forest itself as classifier in the final step, the feature importance measures of the random forest have been found to reduce the amount of features. Most studies using the Gini importance [22,29] and the related permutation-based feature importance of random forests [16,18,20,21,23] together with random forests in a recursive feature elimination scheme, also showed an increases in prediction performance. (Only [17] reports a constant performance, but with greatly reduced amount of features.) While these experiments indicate the efficiency of the Gini importance in an explicit *feature selection *[24] one might raise the question whether a random forest – the "native" classifier of Gini importance – with its orthogonal splits of feature space is optimal also for the *classification *of spectra with correlated features and data-specific noise (Fig. 1), or if other classification models may be a better match with properties of spectral data.

### Objective of this study

Thus, in the present work, we were interested in evaluating the combination of a feature selection by Gini importance together with standard chemometric classification approaches, such as discriminant PCR and PLS classification (D-PCR and D-PLS, respectively) which are known to be well adapted to spectra, and in studying their performance in dependence of specific characteristics of spectral data. In a first experiment we evaluated the joint application of explicit and implicit dimension reduction, using uni- and multivariate feature selection strategies *in combination *with random forest, D-PLS and D-PCR classification in an explicit recursive feature elimination (Table 1). In a second experiment, we studied the influence of different noise processes on random forest and D-PLS classification to identify optimal conditions for explicit and implicit dimension reduction. In both experiments we were interested in identifying general properties and differences of the methods employed in the classification of spectral data.

**Table 1:** Recursive feature selection.

**1.**	**Calculate feature importance **on the training data
	a. Gini importance
	b. absolute value of regression coefficients (PLS/PCR)
	c. p-values from Wilcoxon-test/t-test
**2.**	**Rank the features **according to the importance measure, remove the p% least important
**3.**	**Train the classifier **on the training data
	A. Random forest
	B. D-PLS
	C. D-PCR
	**and apply **it to the test data
**4.**	**Repeat **1.–4. until no features are remaining
**5.**	**Identify the best **feature subset according to the test error

Workflow of the recursive feature selection, and combinations of feature importance measures (1.a-1.c) and classifiers (3.A-3.C) tested in this study. Compare with results in Table 2 and Fig. 4. Hyper-parameters of PLS/PCR/random forest are optimized both in the feature selection (1.) and the classification (3.) step utilizing the training data only. While Gini importance (1.a) and regression coefficients (1.b) have to be calculated within each loop (step 1.–4.), the univariate measures (1.c) have only to be calculated once.

## Results and discussion

### Visualizing feature importance

Measuring feature relevance using the Gini importance is subject to selection bias on factorial data [30]. Splits are more often sought on variables with a higher number of different factors, and a correction of the Gini importance is necessary in such cases [30-32]. Spectral data, except for count data, represent continuous signals, with a distribution of *N *different values for each spectral channel or feature. Each feature will allow the same number of distinct splits in a random forest classification, and, hence, a measurement of the relevance of spectral regions for a specific classification problem will be unaffected by this potential source of bias.

**Figure 2:**
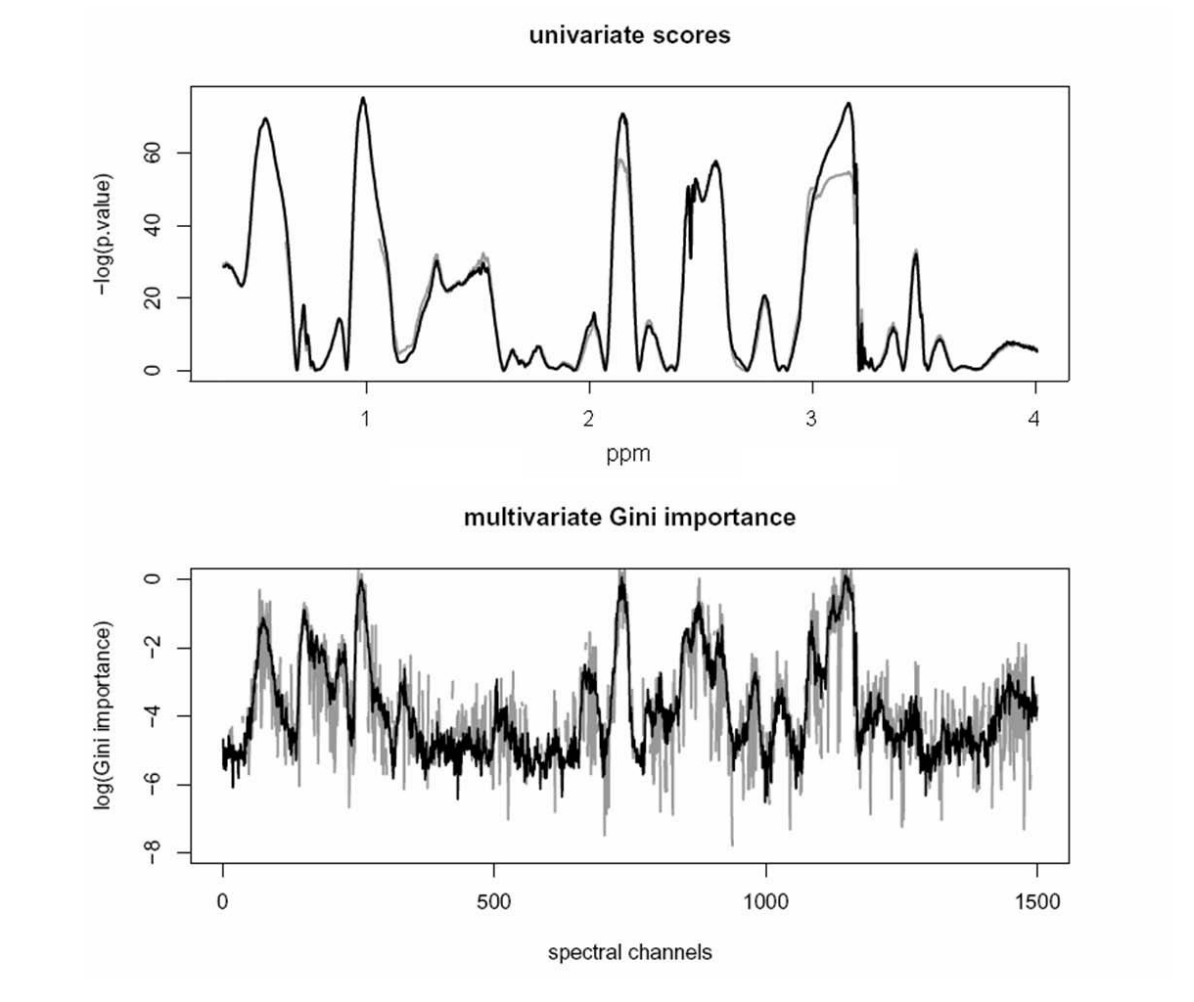
**Importance measures on NMR *candida *data**. in the range from 0.35 to 4 ppm (indicated in the upper figure) for all 1500 spectral channels (indicated in the lower figure). Top: p-values of a t-test (black) and Wilcoxon test (gray). Below: Gini importance of a random forest with 3000 trees (gray) and 6000 trees (black). Compare t ranked measures in Fig. 3.

**Figure 3:**
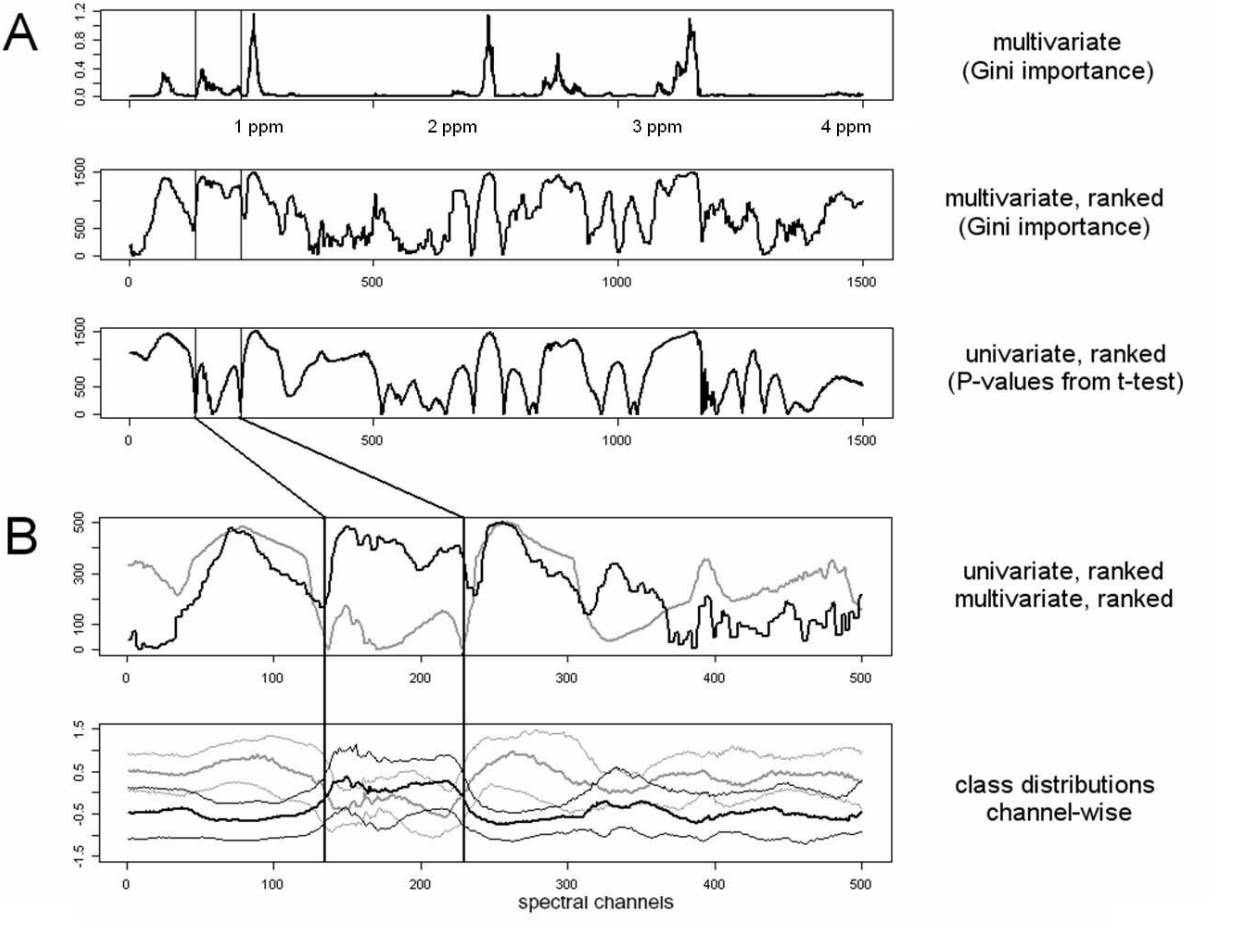
**Comparison of the different feature selection measures applied to the NMR *candida 2 *data (3A)**. Multivariate feature importance measures can select variables that are discarded by univariate measures (3B). Fig. 3A, from top to bottom: Gini importance, absolute values; Gini importance, ranked values, p-values from t-test, ranked values. Fig. 3B: Feature importance scores below (black: Gini importance, gray: t-test). Perhaps surprisingly, regions with complete overlap of the marginal distributions (3B bottom, indicated by vertical lines), are assigned importance by the multivariate measure (3B top). This is indicative of higher-order interaction effects which can be exploited when used as a feature importance measure with a subsequent classifier.

**Figure 4:**
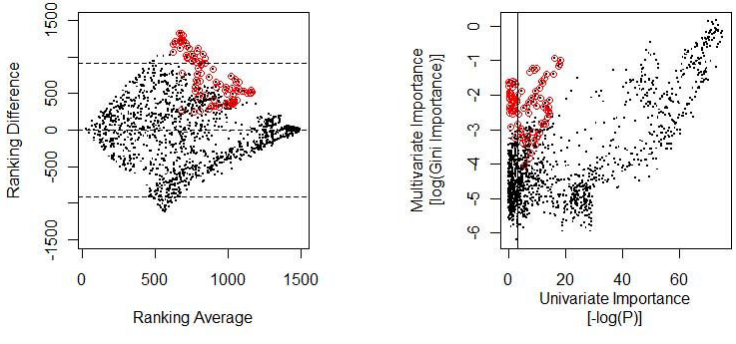
**Tukey mean-difference plot of univariate and multivariate feature importance (left) and correlation of the importance measures shown in Fig. 3A**. Horizontal lines in the left Fig. indicate differences of more than two sigma, the vertical line in the right Fig. indicates a threshold on the univariate P-value of 0.05 (with relevant features being to the right of the vertical line). -. The importances assigned by univariate and multivariate measures are generally highly correlated; many of the features marked in red (corresponding to the spectral channels indicated in Fig. 3B), however, are flagged as uninformative by a univariate measure and as relevant by a multivariate measure.

Both univariate tests for significant class differences returned smooth importance vectors when employed on the spectral data (Fig. 2, top). The smoothness of the Gini importance was dependent on the size of the random forest (Fig. 2, bottom) – small forests resulted in "noisy" importance vectors, only converging towards smooth vectors when increasing the overall number of trees in the forest or the overall number of splits. As such changes influence the absolute value of this measure, the Gini importance could not be interpreted in absolute terms – like the p-values of the univariate tests – but only allowed for a relative comparison. For such a comparison between different variables and between different measures, the features were ranked according to their importance score (Fig. 3A). Here, univariate importance measures and Gini importance agreed well in many, although not all, spectral regions (Fig. 3A, rows 2 and 3). An example of the most prominent differences between univariate feature importance and multivariate Gini importance are highlighted in Fig. 3B. Spectral regions deemed unimportant by the univariate measures – with complete overlap of the marginal distributions as shown in Fig. 3B – may be attributed high importance by the multivariate importance measure (Fig. 4), indicating spectral regions with features of higher order interaction.

Inspecting the Gini feature importance we observed – similar to [32] – that some spectral regions were selected as a whole, suggesting that correlated variables were assigned similar importance. Thus, the importance measure may be interpreted like a spectrum, where neighbouring channels of similar importance may be considered as representatives of the same peak, absorbance or resonance line. This can be used in an exploratory visualization of feature relevance (Figs. 2 and 3A, top row). As the random forest prefers splits on correlated variable over splits on uncorrelated ones [28] it should be noted, however, that this "importance spectrum" may be somewhat biased towards overestimating the importance of major peaks spanning over many spectral channels.

### Feature selection and classification

**Figure 5:**
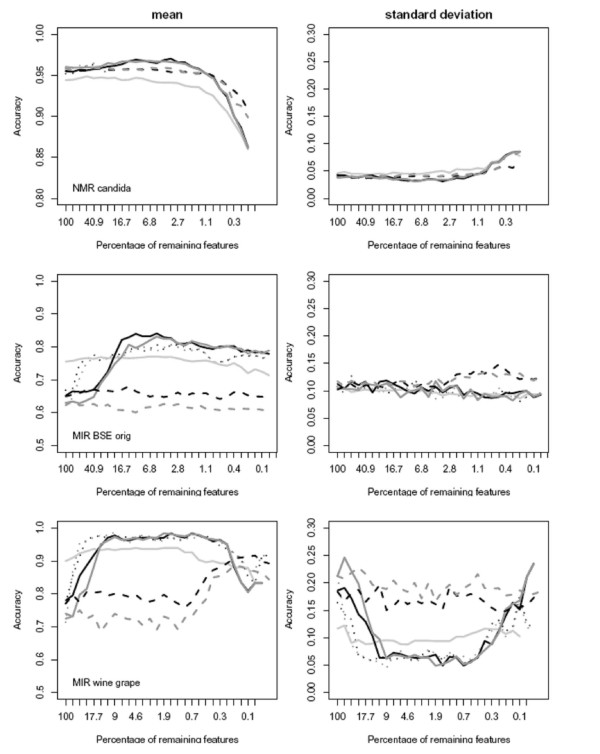
**Classification accuracy (left column) and standard error (right column) during the course of recursive feature elimination for PLS regression (black), PC regression (dark gray) and random forest (light gray), in combination with different feature selection criteria: univariate (dotted), PLS/PC regression (dashed) and Gini importance (solid)**.

The classification accuracies provided by the first experiment based on the real data allowed for a quantitative comparison of the methods applied and for testing for statistically significant differences between results on the full set of features in comparison to the subselected data sets (Table 2, "stars"). On one half of the data, the feature selection hardly changed the classification performance at all (Tables 2 and 3, *tumor *and *candida *data), while on the other half a feature selection improved the final result significantly (Tables 2 and 3, *wine *and *BSE *data), almost independently of the subsequent classifier. In the latter group optimally subselected data typically comprised about 1–10% of the initial features (Table 3, Fig. 5). Such a data dependence in the benefit of a preceding feature selection is well known (e.g. [33]). Different from [33], however, we did not see a relation to the apparent degree of ill-posedness of the classification problem (i.e., a low ratio *N/P *of the length of the spectral vector *P *and the number of available samples *N *leading to an underdetermined estimation problem – he *BSE *and *candida *data, for example, are nearly identical in dimensionality – *P*_*BSE *_= *1209*, *P*_*candida *_= *1500 *– and number of training samples – *N*_*BSE *_= 2 * 96, *N*_*candida *_= 2 * 101).

**Table 2:** Average cross-validated prediction accuracy.

		no selection			Univariate selection			Multivariate selection (Gini importance)			multivariate selection (PLS/PC)		
		
		PLS	PC	RF	PLS	PC	RF	PLS	PC	RF	PLS	PC	RF
MIR BSE	orig	66.8	62.9	74.9	80.7	80.7	76.7	**84.1**	**83.2**	77.4	68	63.5	75.5
		-	-	-	***	***	*	***	***	**	**		
	
	binned	72.7	73.4	75.3	80.4	80.7	76.6	**86.8**	**85.8**	77.3	**85**	82.1	75.6
		-	-	-	***	***	**	***	***	**	***	***	

MIR wine	French	69.5	69.3	**79.3**	**83.7**	**83.5**	**82.2**	**82.4**	**81**	**81.2**	66.9	70.0	**79.8**
		-	-	-	***	**		***	**	*			
	
	grape	77	71.4	90.2	**98.1**	**98.7**	90.3	**98.4**	**98.4**	94.2	91.7	88.5	90.4
		-	-	-	***	***		***	***	**	***	***	

NMR tumor	all	88.8	89	89	89.3	**89.3**	**90.5**	**90.0**	**89.6**	**89.6**	**89.3**	89.2	89.1
		-	-	-	*		***	**		*			
	
	center	71.6	**72.3**	**73.1**	**73.9**	**72.7**	**73.9**	**72.6**	**72.0**	**74.3**	**71.8**	**72.7**	**73.3**
		-	-	-	**			*					

NMR candida	1	**94.9**	**94.6**	90.3	**95.1**	**94.9**	90.6	**95.6**	**95.3**	90.3	**95.3**	**95.2**	90.7
		-	-	-									
	
	2	**95.6**	**95.2**	93.2	**95.8**	**95.7**	93.7	**95.6**	**95.5**	93.5	**96.0**	**95.9**	94.1
		-	-	-							*		
	
	3	93.7	**93.8**	89.7	**93.7**	**93.8**	89.9	**94.2**	**93.8**	89.9	**94.0**	**94.0**	90.2
		-	-	-				*		*	*		
	
	4	86.9	**87.3**	83.9	**87.8**	**87.3**	84.0	**88.2**	**87.6**	84.3	**87.7**	**87.6**	84.1
		-	-	-				*					
	
	5	**92.7**	**92.6**	89.2	**92.7**	**92.6**	89.9	**92.5**	**92.5**	90.3	**92.8**	**92.6**	90.0
		-	-	-									

The best classification results on each data set are underlined. Approaches which do not differ significantly from the optimal result (at a 0.05 significance level) are set in bold type (see methods section). Significant differences in the performance of a method as compared to the same classifier without feature selection are marked with asterisks (* p-value < 0.05, ** p-value < 0.01, *** p-value < .001). The MIR data of this table benefit significantly from a feature selection, whereas the NMR data do so only to a minor extent. Overall, a feature selection by means of Gini importance in conjunction with a PLS classifier was successful in all cases and superior to the "native" classifier of Gini importance, the random forest, in all but one cases.

**Table 3:** Benefit from feature selection.

		univariate selection			multivariate selection (Gini importance)			multivariate selection (PLS/PC)		
		
		PLS	PC	RF	PLS	PC	RF	PLS	PC	RF
MIR BSE	orig	10.0 (6)	10.0 (4)	1.5 (7)	10.0 (6)	10.0 (6)	2.0 (6)	3.0 (13)	0.9 (80)	0.5 (51)
	
	binned	6.0 (5)	7.0 (5)	2.2 (9)	10.0 (9)	10.0 (6)	3.0 (9)	10.0 (5)	9.0 (4)	0.4 (51)

MIR wine	French	4.0 (3)	3.0 (2)	0.7 (64)	5.0 (3)	3.0 (1)	3.0 (26)	0.0 (100)	0.6 (33)	0.0 (64)
	
	grape	8.0 (2)	8.0 (21)	0.6 (64)	10.0 (4)	10.0 (5)	2.0 (11)	4.0 (1)	6.0 (1)	0.0 (64)

NMR tumor	all	1.0 (80)	0.5 (11)	4.0 (6)	4.0 (51)	0.0 (100)	2.0 (6)	0.8 (11)	0.0 (100)	0.3 (80)
	
	center	2.0 (7)	0.4 (6)	0.8 (86)	2.0 (26)	0.2 (64)	0.7 (41)	0.0 (100)	0.7 (13)	0.3 (80)

NMR candida	1	0.5 (80)	0.0 (80)	0.8 (80)	0.0 (100)	0.0 (100)	0.8 (41)	0.4 (64)	0.0 (100)	0.4 (9)
	
	2	0.4 (80)	0.9 (64)	0.0 (80)	2.0 (64)	0.4 (26)	0.0 (100)	2.0 (21)	1.0 (21)	0.4 (41)
	
	3	0.0 (100)	0.0 (100)	0.0 (80)	2.0 (80)	0.6 (80)	2.0 (26)	2.0 (80)	0.0 (100)	0.7 (41)
	
	4	0.8 (80)	0.0 (100)	0.0 (80)	2.0 (80)	1.0 (80)	2.0 (64)	0.7 (33)	0.0 (100)	0.3 (32)
	
	5	0.0 (100)	0.0 (100)	0.7 (80)	0.0 (100)	0.4 (80)	1.0 (64)	0.7 (64)	0.7 (80)	0.4 (21)

Significance of accuracy improvement with feature selection as compared to using the full set of features; and percentage of original features used in a classification that has maximum accuracy (in parentheses). The significance is specified by -log10(p), where p is the p-value of a paired Wilcoxon test on the 100 hold-outs of the cross-validation (see text). For comparison, -log(0.05) = 1.3 and -log(0.001) = 3; the value of 6.0 reported for *MIR BSE binned *in the second row of the first column corresponds to a highly significant improvement in classification accuracy, corresponding to a p-value of 10^-6^.

Random forest, the only nonlinear classifier applied, performed slightly better than the linear classifiers on the unselected data sets (*BSE *and *wine *data, Fig. 5), but improved only moderately in the course of the feature selection (Fig. 5, Table 3: p-value > 10^-3^). Given that random forest performs well on the unselected data sets, and that little or no benefit is incurred by an additional explicit feature selection (Table 2, Fig. 5), it is apparent that an implicit feature selection is at work and performs well when training the random forest classifier. Ultimately, however, the random forest classifier was surpassed in performance by any of the regularized linear methods on all data sets (Table 2: column 9 vs. column 7–8). This rather weak classification performance of the random forest may be seen in line with [20], but contrasts results of e.g. [10,18] using random forest in the classification of microarrays, similar to spectra in their high dimensionality of their feature vectors. Few differences could be observed between D-PLS and D-PCR classification. Among the different feature selection strategies, the Wilcoxon-test and the Gini importance performed better on average than the iterated selection based on the regression coefficients (Fig. 5, Table 2), with slightly better classification results for the Gini importance (Table 2). Overall, while the Gini importance was preferable in feature selection, the chemometric methods performed better than random forest in classification, in spite of their limitation to model linear dependencies only.

**Figure 6:**
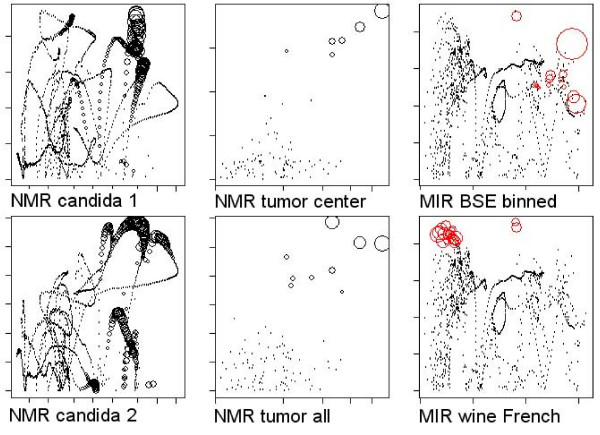
**Channel-wise variance of each feature (horizontal axis) and its correlation with the dependent variable (vertical axis)**. For the data sets of the left and the central column, a feature selection was not required for optimal performance, while the data sets shown in the right columns benefitted from a feature selection. Circle diameter indicates magnitude of the coefficient in the PLS regression. In the right column selected features are shown by red circles, while (the original values of) eliminated features are indicated by black dots. Relevant features show both a high variance and correlation with the class labels.

The two linear classifiers of this study generally seek for subspaces *c*_*k *_maximizing the variance *Var *of the explanatory variables *X *in the subspace *c*



in case of PCR or the product of variance and the (squared) correlation *Corr*



with the response *y *in case of PLS [2,3]. Thus, for a better understanding of D-PCR and D-PLS, both *Corr*(*x*, *y*) and *Var*(*x*) were plotted for individual channels and for individual learning tasks in Fig. 6 (with the absolute value of the coefficients of *c *encoded by the size of the circles in Fig. 6). On data sets which did not benefit greatly from the feature selection, we observed variance and correlation to be maximal in those variables which were finally assigned the largest coefficients in the regression (indicated by the size of the black circles in Fig. 6). Conversely, in data sets where a feature selection was required, features with high variance but only moderate relevance to the classification problem (as indicated by a low univariate correlation or multivariate Gini importance) were frequently present in the unselected data (Fig. 6, black dots). This might be seen as a likely reason for the bad performance of D-PCR and D-PLS when used without preceding feature selection on the *BSE *and *wine *data: Here the selection process allowed to identify those features where variance coincided with class-label correlation (Fig. 6, red circles), leading to a similar situation in the subsequent regression as for those data sets where a feature selection was not required (Fig. 6, compare subselected features indicated red in the left and central row with features in the right row).

In summary, observing that the degree of ill-posedness is not in itself an indicator for a required feature selection preceding a constrained classification, it might be argued that non-discriminative variance – hindering the identification of the optimal subspace in PCR, and disturbing the optimal trade-off between correlation and variation in PLS – may be a reason for the constrained classifiers' failing on the unselected data and, consequently, a requirement for a feature selection in the first place.

### Feature selection and noise processes

The first experiment advocated the use of the Gini importance for a feature selection preceding a constrained regression for some data sets. Thus, and in the light of the unexpectedly weak performance of the random forest classifier, we studied the performance of the D-PLS and the random forest classifier as a function of noise processes which can be observed in spectral data (see Methods section for details) to identify optimal situations for the joint use of explicit and implicit feature selection.

**Figure 7:**
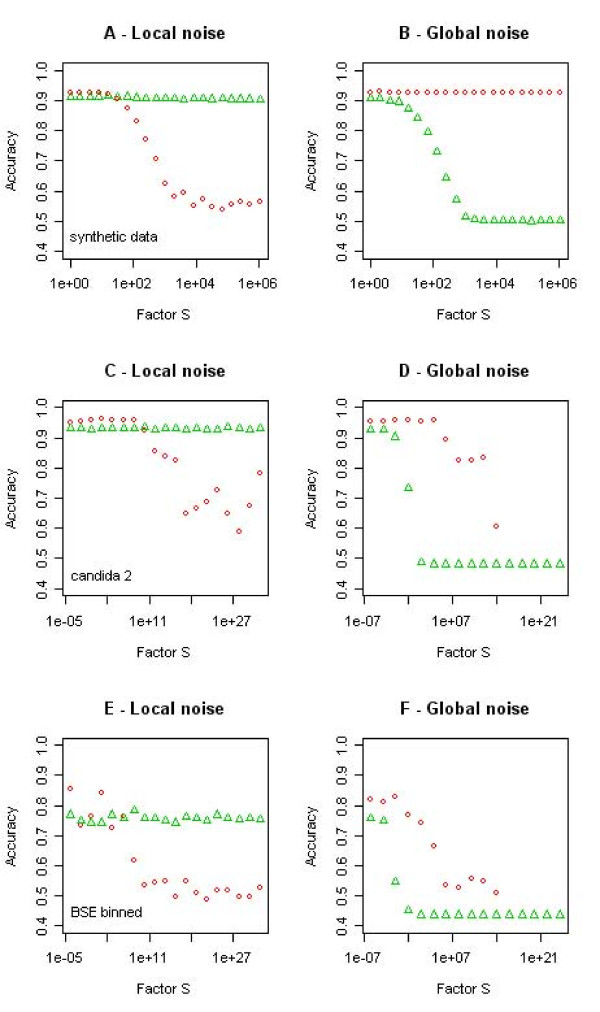
**The effect of different noise processes on the performance of a random forest (green triangles) and a PLS classification (red circles)**. In the left column, feature vectors are augmented by a random variable, which is subsequently rescaled according to a factor S (horizontal axis), thus introducing non-discriminatory variance to the classification problem. In the right column, a random variable scaled by factor S is added as constant offset to the feature vectors, increasing the correlation between features (see text for details). Shown are results on the basis of the bivariate classification problem of Fig. 1 (top row), the *NMR candida 2 *data (middle), and the *BSE binned *data (below).

In this second experiment, random forest proved to be highly robust against the introduction of "local" noise, i.e. against noise processes affecting few spectral channels only, corresponding to spurious peaks or variant spectral regions which are irrelevant to the classification task (both on the synthetic bivariate classification problem, Figs. 1 left, 7A; and the modified real data, Fig. 7CE). The random forest classifier was, however, unable to cope with additive global noise: Already random offsets that were fractions of the amplitude *S *of the spectra (Fig. 7DF; *S *= 10^-2^) resulted in a useless classification by the random forest. As global additive noise stretches the data along the high dimensional equivalent of the bisecting line (Fig. 1), the topology of its base learners may be a disadvantage for the random forest in classification problems as shown in Fig. 1. Single decision trees, which split feature space in a box-like manner orthogonal to the feature direction are known to be inferior to single decision trees splitting the feature space by oblique splits [34] (although they have a considerable computational advantage). Random offsets often occur in spectral data, for example resulting from broad underlying peaks or baselines, or from the normalization to spectral regions that turn out to be irrelevant to the classification problem. Thus, one might argue that the "natural" presence of a small amount of such noise may lead to the rather weak overall performance of the random forest observed in the first experiment (Table 2, Fig. 5).

Partial least squares performed slightly better than random forests on all three data sets at the outset (Fig. 7). In contrast to the random forest, PLS was highly robust against global additive noise: On the synthetic classification problem – being symmetric around the bisecting line – the random offsets did not influence the classification performance at all (Fig. 7B). On the real data – with more complex classification tasks – the D-PLS classification still showed to be more robust against random offsets than the random forest classifier (Fig. 7DF). Conversely, local noise degraded the performance of the D-PLS classification (Fig. 6ACE, although for rather large values of *S *only). The D-PLS classifier seemed to be perfectly adapted to additive noise – splitting classes at arbitrary oblique directions – but its performance was degraded by a large contribution of non-discriminatory variance to the classification problem (Figs. 6 &7ACE).

**Figure 8:**
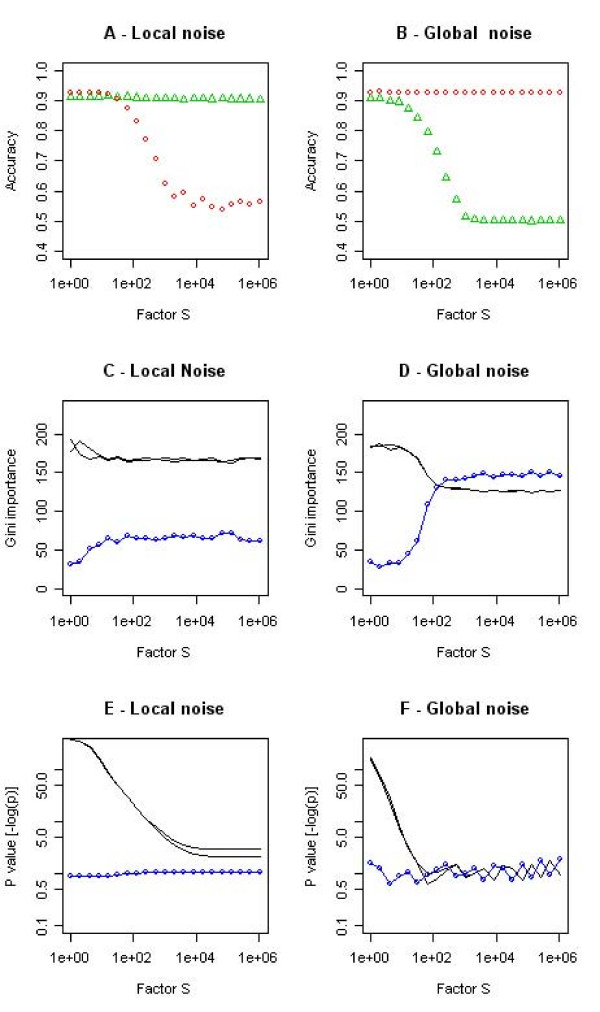
**The effect of different noise processes on the performance of the feature selection methods in the synthetic bivariate classification problem illustrated in Fig. 1**. In the left column feature vectors are extended by a random variable scaled by S, in the right column a random offset of size S is added to the feature vectors. Top row: classification accuracy of the synthetic two-class problem (as in Fig. 7, for comparison); second row: multivariate Gini importance, bottom row: p-values of univariate t-test. The black lines correspond to the values of the two features spanning the bivariate classification task (Fig. 1), the blue dotted line corresponds to the third feature in the synthetic data set, the random variable. The performance of the random forest remains nearly unchanged even under the presence of a strong source of "local" noise for high values of S.

In the presence of increasing additive noise, both univariate and multivariate (i.e., the Gini importance) feature importance measures lost their power to discriminate between relevant and random variables at the end (Fig. 8DF), with the Gini importance retaining discriminative power somewhat longer finally converging to a similar value for all three variables correlating well with a random classification and an (equally) random assignment of feature importance (Fig. 8D). When introducing a source of local random noise and normalizing the data accordingly, the univariate tests degraded to random output (Fig. 8E), while the Gini importance measure (Fig. 8CE) virtually ignored the presence and upscaling of the non-discriminatory variable (as did the random forest classifier in Fig. 7ACE).

### Feature selection using the Gini importance

Overall, we observed that the random forest classifier – with the non-oblique splits of its base learner – may not be the optimal choice in the classification of spectral data. For feature selection, however, its Gini importance allowed to rank non-discriminatory features low and to remove them early on in a recursive feature elimination. This desirable property is due to the Gini importance being based on a rank order measure which is *invariant to the scaling *of individual variables and unaffected by non-discriminatory variance that does disturb D-PCR and D-PLS. Thus, for a constrained classifier requiring a feature selection due to the specificities of the classification problem (Table 2, Fig. 5), the Gini feature importance might be a preferable ranking criterion: as a *multivariate *feature importance, it is considering conditional higher-order interactions between the variables when measuring the importance of certain spectral regions, providing a better ranking criterion than a univariate measure used here and in similar tasks elsewhere [9,10].

A comparison of the computing times of the different feature selection and classification approaches (Table 4) shows that the computational costs for using the Gini importance is comparable to the cost of using the other multivariate feature selection criterion tested in this study. On average the computing time was no more than twice as long as for the more basic univariate importance measures.

**Table 4:** Computing times.

		no selection			univariate selection			Multivariate selection (Gini importance)			multivariate selection (PLS/PC)		
		
		PLS	PC	RF	PLS	PC	RF	PLS	PC	RF	PLS	PC	RF
MIR BSE	orig	5.7	11.1	9.9	46.4	53.9	46.8	88.8	97.0	91.5	87.9	92.4	88.0
	
	binned	2.8	3.2	3.1	13.6	14.7	15.9	26.1	27.1	29.0	28.7	29.6	31.5

MIR wine	French	8.8	7.8	2.4	26.6	21.8	7.7	47.0	45.9	33.5	17.2	14.7	7.4
	
	grape	12.1	10.3	2.5	28.9	22.3	8.0	54.0	47.6	33.5	15.8	13.1	6.5

NMR tumor	all	0.3	0.4	0.4	1.4	1.2	2.1	2.9	2.7	3.6	3.6	3.4	4.3
	
	center	0.2	0.2	0.2	1.1	0.8	1.1	2.2	1.9	2.1	2.1	1.8	2.0

NMR candida	1	4.6	8.8	7.7	22.4	41.2	37.1	43.5	62.5	61.1	59.8	78.4	75.4
	
	2	3.7	4.8	3.8	18.0	22.0	19.4	34.5	38.5	37.3	36.3	40.3	37.9
	
	3	3.7	4.7	3.7	17.4	20.1	17.9	33.4	36.0	34.7	34.6	37.8	35.1
	
	4	3.9	5.1	4.8	18.7	23.4	24.3	36.0	40.5	60.5	41.6	46.2	47.0
	
	5	3.5	3.9	2.6	31.9	32.4	27.0	62.6	63.0	60.0	58.3	43.4	38.5

The table reports the runtime for the different feature selection and classification approaches, and the different data sets (on a 2 GHz personal computer with 2 GB memory). Values are given in minutes, for a ten-fold cross-validation and with parameterisations as used for the results shown in Tables 2 and 3. For all methods, a univariate feature selection takes about five times as long as a classification of the same data set without feature selection. Both multivariate feature selection approaches require approximately the same amount of time for a given data set and classifier. Their computing time is no more than twice as long as in a recursive feature elimination based on a univariate feature importance measure.

## Conclusion

In the joint application of the feature selection and classification methods on spectral data neither the random forests classifier using the Gini importance in a recursive feature selection, nor a constrained regression without feature selection were the optimal choice for classification. Random forest showed to be robust against single noisy features with a large amount of non-discriminatory variance. Unfortunately it also showed to be highly sensible to random offsets in the feature vector, a common artefact in spectral data. D-PLS was capable of dealing with such offsets, although it failed in the presence of non-discriminatory variance in single, highly variable features. The removal of such irrelevant – or even misleading – predictors was crucial in the application of the constrained classifiers tested in this study. Overall, the combined application of Gini importance in a recursive feature elimination together with a D-PLS classification was either the best approach or – in terms of statistical significance – comparable to the best in all classification tasks, and may be recommended for the separation of binary, linearly separable data.

The results also suggest that when using a constrained learning method – such as the D-PLS or D-PCR classifier as in this study – the main purpose of a feature selection is the removal of few "noisy" features with a large amount of variance, but little importance to the classification problem. Then, the feature elimination is a first step in the regularization of a classification task, removing features with non-discriminatory variance, and allowing for a better regularization and implicit dimension reduction by the subsequent classifier. Considering the similarity of PLS, ridge regression and continuum regression [2,3,35] – all of them trading correlation with class labels, and variance of the data for a regularization – one might expect this to be a general feature for these constrained regression methods.

Only binary classifications tasks were studied here, but one may expect that results generalize to multi-class problems as well when using, for example, penalized mixture models in place of a D-PLS classification. It might be worthwhile to test whether using a constrained classifier in the final classification step of a recursive feature selection is able to increase the classification performance on other data as well, for example on microarrays where a recent study [20] reported of a general advantage of support vector machines with RBF-kernel over the random forest classifier.

Of course, rather than advocating a hybrid method using random forest for feature selection and a constrained linear classifier to predict class membership, it might be advantageous to adapt the random forest classifier itself to fit the properties of spectral data in an optimal fashion. For individual tree-like classifiers, a large body of literature about trees using such non-orthogonal, linear splits in their nodes is available [34] and may be used for such an adaption of the random forest classifier.

## Methods

In a first experiment, we systematically evaluated the joint use of different feature selection and classification methods, on a number of different spectral data sets. In a second, we looked into the behaviour of the random forest and D-PLS classifier on synthetically modified data, to understand specific properties of these methods when applied to spectra.

### Experiment 1: Joint feature selection and classification

In general, feature selection is a concern in both regression (prediction of a continuous response) and in classification (prediction of two or more categories). Here, we confined ourselves to binary classification tasks. Our experiments were based on four different data sets available to us from different studies [36-39], providing – with different preprocessing, labels, or dichotomous sub-problems – eleven binary classification tasks (Table 2).

#### Classification and feature selection methods

Three different feature selection approaches were applied to the data in an explicit recursive feature elimination (Table 1), together with the three following classifiers: linear discriminant principal component (D-PCR) and partial least squares (D-PLS) classification (using [40]) and the nonlinear random forest classifier (RF) (using [41]). While trees in the random forest allow for a classification via majority votes and a binary decision, D-PCR and D-PLS classification were used with a predefined and fixed threshold, i.e. a score of 0.5 intermediate to the trained class values 0 and 1, using balanced classes during training and for the test (see below).

As univariate feature selection measure, the p-values of channel-wise Wilcoxon-tests for class differences were used to rank the features and to allow for a filtering of features prior to the D-PLS and D-PCR classification. In the two multivariate feature selection procedures applied, variables were recursively eliminated either according to smallest PLS or PC regression coefficient (as in [5], although without stability test), or according to smallest Gini importance value. In the latter case the actual classification in the selected subspace was performed not only by a RF as in [15-18], but also by the linear classifiers. In total, seven different combinations of feature selection and classification methods were applied to the data (Table 2). The classification of the data without any feature selection was tested as well. For the results shown in the last column of Tables 2 and 3 – i.e. the combination of feature selection using regression coefficients and a subsequent classification by RF – PLS was used in feature selection. For the sake of computational simplicity, all multivariate feature selection measures were optimized using their own cost function and not in a joint loop with the subsequent classifier (using a cross-validated least-squares-error for PLS and PCR regression coefficients and the out-of-bag classification error of the RF for Gini importance, optimized over the same parameter spaces as the respective classifiers). For both univariate filters and multivariate wrappers, 20% of the remaining features were removed in each iteration step. Prior to classification all data was subject to L_1 _normalization, i.e. to a normalization of the area under the spectrum in a predefined spectral region.

#### Data

Data set one, the *BSE *data set, originates from a study concerning a conceivable ante-mortem test for bovine spongiform encephalopathy (BSE). Mid-infrared spectra of *N = 200 *dried bovine serum samples (*N*_*pos *_= *95*, *N*_*neg *_= *105*) were recorded in the spectral range of 400–4000 *cm*^-1 ^with *P = 3629 *data points per spectrum. Details of the sample preparation and of the acquisition of spectra are reported in Refs. [14,31]. The same spectra were used in a second binary classification task after a smoothing and downsampling ("binning"), and thus by reducing the number of data points per spectrum to *P*_*red *_= *1209*.

Data set two, the *wine *data set, comprised *N = 71 *mid-infrared spectra with a length of *P = 3445 *data points from the spectral region of 899–7496 *cm*^-1^, sampled at a resolution of approx. 4 *cm*^-1 ^interpolated to 2 *cm*^-1^, originating from the analysis of 63 different wines using an automated MIRALAB analyzer with AquaSpec flow cell. In the preprocessing a polynomial filter (Savitzky-Golay, length 9) of second order was applied to the spectra. Labels assigned to these data were the type of grape (*N*_*red *_= *30*, *N*_*white *_= *41*) in a first learning task and an indicator of the geographic origin of the wine (*N*_*French *_= *26*, *N*_*World *_= *45*) in a second.

Data set three, the *tumor *data set, comprised *N = 278 *in vivo 1H-NMR spectra with a length of *P = 101 *data points from the spectral region between approximately 1.0 *ppm *and 3.5 *ppm*, originating from 31 magnetic resonance spectroscopic images of 31 patients, acquired at 1.5 T with an echo time of 135 *ms *in the pre-therapeutic and post-operative diagnostics of (recurrent) brain tumor (*N*_*healthy *_= *153*, *N*_*tumor border *_= *72, N*_*tumor center *_= *53*) [31,32]. Two binary groupings were tested, either discriminating healthy vs. both tumor groups (*tumor all*), or the spectral signature of the tumor center vs. the remaining spectra (*tumor center*).

Data set four, the *candida *data set, comprised *N = 581 *^1^H-NMR spectra of cell suspensions with a length of *P *= 1500 data points in between 0.35–4 ppm, originating from a chemotaxonomic classification of yeast species (Candida albicans, C. glabrata, C. krusei, C. parapsilosis, and C. tropicalis). A subset of the data was originally published in [34]. Its five different subgroups of sizes *N = {175, 109, 101, 111, 85} *allowed to define five different binary subproblems ("one-against-all").

#### Comparison

In the evaluation, 100 training and 100 test sets were sampled from each of the available data sets, in a ten times repeated ten-fold cross validation [42,43], following the overall test design in [19]. In order to obtain equal class priors both in training and testing, the larger of the two groups of the binary problems was subsampled to the size of the smaller if necessary. Where dependence between observations was suspected, e.g. in the *tumor *data where more than one spectrum originated for each patient, the cross-validation was stratified to guarantee that all spectra of a correlated subset were exclusively assigned to either the training or the test data [42,43].

The random forest parameters were optimized in logarithmic steps around their default values [41] (using 300 trees, and a random subspace with dimensionality equal to the rounded value of the square of the number of features) according to the out-of-bag error of the random forest, while the number of latent variables *γ *in the linear classifiers was determined by an internal five-fold cross-validation for each subset, following the 1*σ *rule for choosing the *γ *at the intersection between the least error (at *γ*_*opt*_) plus an interval corresponding to the 1*σ *standard deviation at *γ*_*opt*_, and the mean accuracy.

The classification accuracy was averaged over all 100 test results and used as performance measure in the comparison of the different methods. While all feature selection and all optimization steps in the classification were performed utilizing the training data only, test result were recorded for all feature subsets obtained during the course of feature selection (Fig. 4). To verify significant differences between the test results, a paired Cox-Wilcoxon test was used on the accuracies of the 100 test sets as proposed in [42,43]. Such paired comparisons were performed for each classifier between the classification result obtained for the full set of features, and the best result when applied in conjunction with a selection method (i.e. the results with highest classification accuracy in the course of feature selection). Feature selection approaches leading to a significant increase in classification performance were indicated accordingly (Table 2, indicated by stars). Once the best feature selection and classification approach had been identified for a data set (as defined by the highest classification accuracy in a row in Table 2), it was compared against all other results on the same data set. Results which were indistinguishable from this best approach (no statistical difference at a 5% level) were indicated as well (Table 2, indicated by bold values).

### Experiment 2: PLS and RF classification as a function of specific data properties

D-PLS reportedly benefits from an explicit feature selection on some data sets [33]. Random forest reportedly performed well in classification tasks with many features and few samples [15-18], but was outperformed by standard chemometrical learning algorithms when used to classify spectral data. Thus, to identify reasons for these differences and to corroborate findings from experiment one, we decided to study the performance of both methods in dependence of two noise processes which are specific to spectral data.

#### Noise processes

We identified two sources of unwanted variation (noise processes) which can be observed in spectral data, and which can jeopardize the performance of a classifier.

First, there are processes affecting few, possibly adjacent, spectral channels only. Examples for such changes in the spectral pattern are insufficiently removed peaks and slight peak shifts (magnetic resonance spectroscopy), the presence of additional peaks from traces of unremoved components in the analyte, or from vapour in the light beam during acquisition (infrared spectroscopy). Identifying and removing spectral channels affected by such processes is often the purpose of explicit feature selection. We refer to this kind of noise as "local noise" in the following, where the locality refers to the adjacency of channels along the spectral axis.

Second, there are processes affecting the spectrum as a whole. Examples for such noise processes may be the presence (or absence) of broad baselines, resulting in a random additional offset in the spectrum. Variation may also result from differences in the signal intensities due to changes in the concentration of the analyte, variation in reflectance or transmission properties of the sample (infrared spectroscopy), or the general signal amplitude from voxel bleeding and partial volume effects (magnetic resonance spectroscopy), leading to a scaling of the spectrum as a whole, and – after normalization – to random offsets in the spectrum. Such processes increase the nominal correlation between features and are the main reason for the frequent use of high-pass filters in the preprocessing of spectral data (Savitzky-Golay filter, see above). It might be noted that this noise does not have to offset the spectrum as a whole to affect the classification performance significantly, but may only modify those spectral regions which turn out to be relevant to the classification task. Nevertheless, we refer to this kind of noise as "global noise" here.

#### Modified and synthetic data sets

For visualization (Fig 1, left), we modelled a synthetic two-class problem, by drawing 2*400 samples from two bivariate normal distributions (centred at (0,1) and (1,0), respectively, with standard deviation 0.5). The two features for the two-dimensional classification task were augmented by a third feature comprising only random noise (normally distributed, centred at 0, standard deviation 0.5), resulting in a data set with N = 800, P = 3 and balanced classes. To mimic local noise, we rescaled the third, random feature by a factor S, for S = 2^{0,1,...,20}^. In real spectra one might expect S – the ratio between the amplitude of a variable that is relevant to the classification problem, and a larger variable introducing non-discriminatory variance only – to be of several orders of magnitude. Here, changing S gradually increased the variance of the third feature and the amount of non-discriminatory variance in the classification problem. To mimic global noise, we added a constant offset (normally distributed, centred at 0, standard deviation 0.5), also scaled by S = 2^{0,1,...,20}^, to the features of every sample as an offset. This increased the correlation between the features and, along S, gradually stretched the data along the bisecting line (Fig. 1, right).

In addition to the synthetic two-class problem of Fig. 1, we modified two exemplary real data sets (*candida 2 *and *BSE binned*) by these procedures in the same way, here using S = 10^{-6,-4,...,16}^, using the largest amplitude of a spectrum as reference for a shift by S = 1, or a rescaling of the random feature (N(0,.5)).

#### Comparison

Gini importance and univariate importance (t-test) were calculated along S for the features of the synthetic data set. PLS and random forest classification were applied to all data sets, for all values of S, after a L_2_-normalization of the feature vector. (Which may be a closer match with the noise statistic than the L_1 _normalization used in the real data in the first experiment.) Classification accuracy was determined according to the procedure described above (Experiment 1).

## Authors' contributions

BHM performed the design of the study and drafted the manuscript. FAH contributed significantly to the manuscript. RM, UH, PB, WP acquired data for the study. All authors participated in the analysis of the results. BHM, BMK, WP, FAH conceived of the study and participated in its coordination. All authors read and approved the final manuscript.
